# The cadherin domains and the kinesin-binding intracellular domain of CASY-1/calsyntenin function in a redundant manner for learning

**DOI:** 10.17912/micropub.biology.001600

**Published:** 2025-05-16

**Authors:** Hayao Ohno, Yuzuha Komachiya, Yuichi Iino

**Affiliations:** 1 Department of Chemical and Biological Sciences, Faculty of Science, Japan Women's University, Tokyo, Tokyo, Japan; 2 Department of Biological Sciences, School of Science, The University of Tokyo, Tokyo, Tokyo, Japan

## Abstract

Taste avoidance learning in *Caenorhabditis elegans* is regulated by the calsyntenin/alcadein homolog CASY-1 , which transports the insulin receptor DAF-2c to the synaptic region. This transport involves binding of the CASY-1 intracellular domain to the kinesin-1 (KIF5) complex. However, a previous study showed that the intracellular domain of CASY-1 is dispensable for learning. To investigate how CASY-1 functions, we performed functional domain mapping of CASY-1 . Both the cadherin domains of CASY-1 and its binding to kinesin-1 are individually dispensable, while simultaneous loss of both abolished the CASY-1 function, suggesting that CASY-1 enables robust intracellular transport through physical interactions with multiple proteins.

**Figure 1. The interaction between CASY-1 and kinesin-1 is involved in the synaptic DAF-2c localization:**
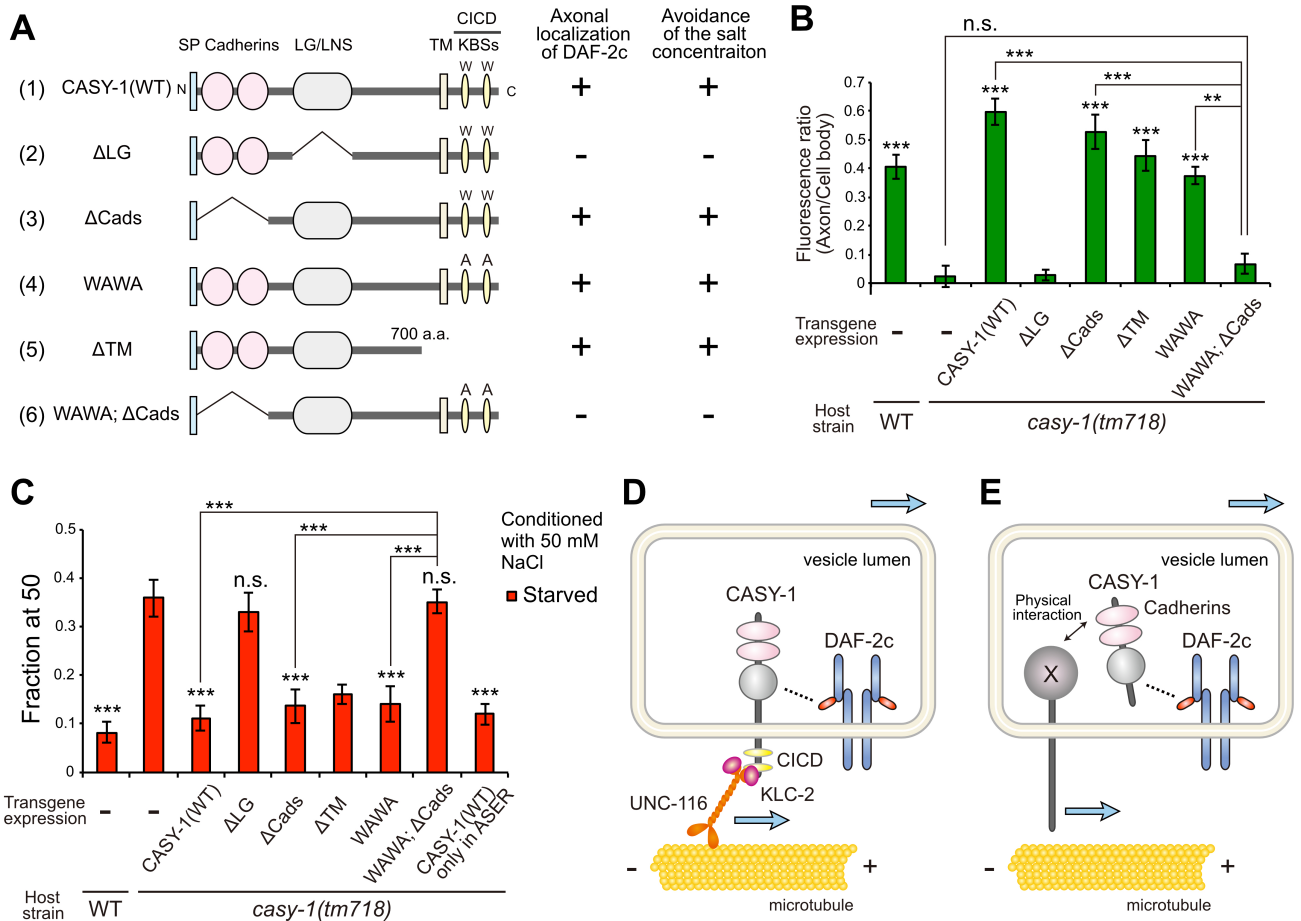
( **A** ) Schematic depiction and functions of the mutated variants of CASY-1 . SP, signal peptide; Cadherins, Cadherin domains; LG/LNS, LG/LNS-like domain; TM, transmembrane region; KBSs, kinesin binding segments; CICD, CASY-1 intracellular C-terminal domain. ( **B** ) Axonal localization of DAF-2c::Venus was quantified in ASER of wild type and *casy-1* mutants transformed with the mutated variants of *casy-1* that are shown in (A). *n* ≥ 9 animals. Difference from *casy-1 ( tm718 )* animals carrying only the transformation marker (−) unless otherwise indicated. *** *p* < 0.001, ** *p* < 0.01 (ANOVA with Tukey's post-test). n.s., not significant. ( **C** ) The salt concentration preference assay was performed on wild type and *casy-1* mutants transformed with the mutant constructs of *casy-1* that are shown in (A). *n* = 21. Difference from *casy-1 ( tm718 )* animals carrying only the transformation marker (−) unless otherwise indicated; *** *p* < 0.001 (ANOVA with Tukey's post-test). n.s., not significant. (B and C) The *casy-1* promoter was used to express transgenes unless otherwise noted. The *gcy-5* promoter was used to express the *casy-1* transgene only in ASER in (C). ( **D** and **E** ) A possible model for the mechanisms by which CASY-1 regulates the axonal DAF-2c localization. CASY-1 interacts with the kinesin-1 complex through the intracellular domain (D) and with an unknown axonal protein(s) through the cadherin domains (E), to be transported to the axon together with DAF-2c.

## Description

The nematode *Caenorhabditis elegans* can memorize external salt concentrations and modify the preference for them according to past experiences; they are attracted to the past salt concentration where they had been fed, whereas they avoid it if they were starved (Kunitomo et al., 2013; Ohno et al., 2014). We found that the avoidance of the salt concentrations associated with starvation was impaired in mutants of *casy-1* (Ikeda et al., 2008; Ohno et al., 2014). CASY-1 is the sole *C. elegans* ortholog of calsyntenins/alcadeins, a family of transmembrane proteins highly expressed in neurons. CASY-1 has the extracellular cadherin and LG/LNS domains and the intracellular kinesin-1 binding segments (KBSs) (Ikeda et al., 2008, Fig. 1A ). It has been suggested that CASY-1 functions in intracellular trafficking and cell adhesion through physical interactions with BAM-2 /neurexin-related (Kim & Emmons, 2017), UNC-104 /kinesin-3 (KIF1) (Thapliyal et al., 2018), and KLC-2 /kinesin-1 (KIF5) light chain (Ohno et al., 2014).

In a previous study (Ohno et al., 2014), we reported that CASY-1 induces starvation-associated learning by acting as an adaptor linking the insulin receptor isoform DAF-2c and the kinesin-1 complex to transport DAF-2c to the synaptic region, based on the following results: (1) CASY-1 physically interacts with KLC-2 and DAF-2c, (2) mutation or knockdown of *unc-116* /kinesin-1 heavy chain or *klc-2* inhibits the synaptic DAF-2c localization, and (3) MAPK-dependent KLC-2 S452 phosphorylation prevents physical interaction between CASY-1 and KLC-2 and this phosphorylation also prevents the synaptic DAF-2c localization. However, another study (Ikeda et al., 2008) found that the expression of the extracellular domain of CASY-1 , which lacks intracellular KBSs, is sufficient to restore the learning defects of *casy-1* mutants.

To understand how CASY-1 regulates the DAF-2c transport and learning, we examined the functions of mutated variants of CASY-1 ( Fig. 1A ) by transgenic rescue experiments, in which the constructs were expressed under the *casy-1* promoter in the *casy-1 ( tm718 )* deletion mutant strain. Consistent with the previous study (Ikeda et al., 2008), deletion of the central LG/LNS domain (ΔLG) abolished the functionality of CASY-1 , whereas that of the cadherin domains (ΔCads) did not, in both the regulation of DAF-2c localization and the starvation-associated learning ( Fig. 1A (2)(3), Fig. 1B, and Fig. 1C ). Interestingly, replacement of the tryptophans of both kinesin-binding motifs in KBSs by alanines (WAWA), which completely disrupts the binding of the intracellular domain of CASY-1 with KLC-2 (Ohno et al., 2014), did not affect the functions of CASY-1 ( Fig. 1A (4), Fig. 1B, and Fig. 1C ). Even the extracellular domain alone (ΔTM), which is assumed to be released from the cell surface, was functional ( Fig. 1A (5), Fig. 1B, and Fig. 1C ). By contrast, the construct that harbors both the replacement of the tryptophans by alanines and the deletion of cadherin domains (WAWA; ΔCads) failed to rescue the *casy-1* mutant phenotypes ( Fig. 1A (6), Fig. 1B, and Fig. 1C ), suggesting that the cadherin domains and the kinesin binding intracellular domain function in a redundant fashion. These results may be explained by assuming that CASY-1 mediates the transport of DAF-2c via two interactions, the interaction of the intracellular domain with kinesin-1 ( Fig. 1D ) and the interaction of the extracellular cadherin domains with an unknown axonal protein(s), which is itself transported probably by interaction with motor proteins ( Fig. 1E ). Since the point mutations in kinesin-1 binding motifs of CASY-1 abolished the functions of the cadherin domains-deleted construct ( Fig. 1A (6)) and the expression of a gain-of-function form of KLC-2 increased the axonal DAF-2c localization in a CASY-1-dependent fashion (Ohno et al., 2014), the association of CASY-1 with kinesin-1 appears to have a significant contribution to the axonal transport of DAF-2c.

## Methods

Salt concentration preference assay was performed as described (Ohno et al., 2014). Young adult worms were exposed to 50 mM NaCl without food for 4 h followed by a behavioral test in which five to nine worms were transferred to an agar assay plate (56 mm [w] x 38 mm [d] plastic dish poured with 4 mL of 2% agar, 5 mM potassium phosphate [pH 6.0], 1 mM CaCl _2_ , 1 mM MgSO _4_ ) with a gradient of NaCl, covering a concentration range of approximately 20 mM to 80 mM. Worms that moved to the ~50 mM (the central half area of an assay plate) area were classified as “50”.

Axonal localization of DAF-2c::venus was evaluated as described (Ohno et al., 2014). Fluorescence was observed with a Leica HCX PL APO 63×/1.30 objective on a Leica TCS-SP5 confocal microscope. Images were analyzed with the software attached to the confocal microscope (LAS AF ver 2.2).

Statistic analyses were performed with a statistic package (Prism v.5, GraphPad software).

## Reagents

**Table:** 

JN1505	*peEx1505* [P *myo-3 ::venus* ].
JN1527	*casy-1 ( tm718 ) II* ; *peEx1527* [P *myo-3 ::venus* ].
JN1528	*casy-1 ( tm718 ) II* ; *peEx1528* [P *casy-1 :: casy-1* ; P *myo-3 ::venus* ].
JN1529	*casy-1 ( tm718 ) II* ; *peEx1529* [P *casy-1 :: casy-1 (∆LG)* ; P *myo-3 ::venus* ].
JN1530	*casy-1 ( tm718 ) II* ; *peEx1530* [P *casy-1 :: casy-1 (∆Cads)* ; P *myo-3 ::venus* ].
JN1531	*casy-1 ( tm718 ) II* ; *peEx1531* [P *casy-1 :: casy-1 (∆TM)* ; P *myo-3 ::venus* ].
JN1532	*casy-1 ( tm718 ) II* ; *peEx1532* [P *casy-1 :: casy-1 (WAWA)* ; P *myo-3 ::venus* ].
JN1533	*casy-1 ( tm718 ) II* ; *peEx1533* [P *casy-1 :: casy-1 (WAWA; ∆Cads)* ; P *myo-3 ::venus* ].
JN1538	*casy-1 ( tm718 ) II* ; *peEx1538* [P *gcy-5 :: casy-1* ; P *myo-3 ::venus* ].
JN1560	*casy-1 ( tm718 ) II* ; *peIs1524* [P *gcy-5 ::daf-2c::Venus* ; P *unc-122 ::mCherry* ]; *peEx1560* [P *casy-1 :: casy-1* ; P *gcy-5 ::mCherry* ; P *lin-44 ::gfp* ].
JN1561	*casy-1 ( tm718 ) II* ; *peIs1524* [P *gcy-5 ::daf-2c::Venus* ; P *unc-122 ::mCherry* ]; *peEx1561* [P *casy-1 :: casy-1 (∆LG)* ; P *gcy-5 ::mCherry* ; P *lin-44 ::gfp* ].
JN1562	*casy-1 ( tm718 ) II* ; *peIs1524* [P *gcy-5 ::daf-2c::Venus* ; P *unc-122 ::mCherry* ]; *peEx1562* [P *casy-1 :: casy-1 (∆Cads)* ; P *gcy-5 ::mCherry* ; P *lin-44 ::gfp* ].
JN1563	*casy-1 ( tm718 ) II* ; *peIs1524* [P *gcy-5 ::daf-2c::Venus* ; P *unc-122 ::mCherry* ]; *peEx1563* [P *casy-1 :: casy-1 (∆TM)* ; P *gcy-5 ::mCherry* ; P *lin-44 ::gfp* ].
JN1564	*casy-1 ( tm718 ) II* ; *peIs1524* [P *gcy-5 ::daf-2c::Venus* ; P *unc-122 ::mCherry* ]; *peEx1564* [P *casy-1 :: casy-1 (WAWA)* ; P *gcy-5 ::mCherry* ; P *lin-44 ::gfp* ].
JN1565	*casy-1 ( tm718 ) II* ; *peIs1524* [P *gcy-5 ::daf-2c::Venus* ; P *unc-122 ::mCherry* ]; *peEx1565* [P *casy-1 :: casy-1 (WAWA; ∆Cads)* ; P *gcy-5 ::mCherry* ; P *lin-44 ::gfp* ].
